# Dye-Sensitized Solar Cell for Building-Integrated Photovoltaic (BIPV) Applications

**DOI:** 10.3390/ma14133743

**Published:** 2021-07-04

**Authors:** Marek Szindler, Magdalena Szindler, Aleksandra Drygała, Krzysztof Lukaszkowicz, Paulina Kaim, Rafał Pietruszka

**Affiliations:** 1 Scientific and Didactic Laboratory of Nanotechnology and Material Technologies, Faculty of Mechanical Engineering, Silesian University of Technology, Towarowa 7 Str., 44-100 Gliwice, Poland; 2 Department of Engineering Materials and Biomaterials, Silesian University of Technology, Konarskiego 18a Str., 44-100 Gliwice, Poland; magdalena.szindler@polsl.pl (M.S.); aleksandra.drygala@polsl.pl (A.D.); krzysztof.lukaszkowicz@polsl.pl (K.L.); paulina.kaim@polsl.pl (P.K.); 3 Institute of Physics, Polish Academy of Sciences, 02-668 Warsaw, Poland; pietruszka@ifpan.edu.pl

**Keywords:** renewable energy, nanotechnology, building-integrated photovoltaics (BIV), thin films, dye-sensitized solar cells

## Abstract

One of the important research directions in the field of photovoltaics is integration with construction. The integration of solar cell systems with a building can reduce installation costs and help optimize the used space. Among the few literature reports on photovoltaic roof tiles, solutions with silicon and thin film solar cells dominate. An interesting solution may be the application of dye-sensitized solar cells. In addition to their interesting properties, they also have aesthetic value. In the classic arrangement, they are constructed using glass with a transparent conductive layer (TCL). This article describes replacing a classic glass counter electrode with an electrode based on a ceramic tile and nickel foil. First, a continuous and homogeneous fluorine-doped tin oxide (FTO) thin film was developed so that the above-mentioned substrate could be applied. The atomization method was used for this purpose. Then, nanocolloidal platinum paste was deposited as a catalytic material using the screen printing method. The electrical parameters of the manufactured DSSCs with and without a counter electrode tile were characterized by measuring their current–voltage characteristics under standard AM 1.5 radiation. A dye-sensitized solar cell integrated with ceramic tiles and nickel foil was produced and displayed an efficiency of over 4%. This solution makes it possible to expand their construction applications. The advantage of this solution is full integration with construction, while simultaneously generating electricity. A dye-sensitized solar cell was built layer-by-layer on a ceramic tile and nickel foil.

## 1. Introduction

Photovoltaics is a field covering the issues of obtaining electricity from sunlight, as well as its processing and storage [1]. The first efficient (6%) crystalline silicon solar cells were made by Chapin, Fuller, and Pearson of Bell Laboratories in 1953 by diffusing boron into n-type silicon [2]. Five years later, Maldenkorn and colleagues at the United States Army Electronics Research and Development Laboratories in Belmar, NJ, developed n-p solar cells. In the summer of 1960, RCA Laboratories showed that these solar cells were much more resistant to cosmic rays than p-n cells, which led to extremely intense research and development into silicon solar cells for use as energy sources in spacecraft [1,2]. During the 1970s energy crisis, the idea of also using solar cells for terrestrial purposes began, which made work related to the improvement of solar cell technology one of the most attractive research directions [3]. In the 1970s, PV modules were initially installed on buildings where there was no access to traditional electricity [4]. It was not until the 1980s that the first modules mounted on the roofs of single-family houses were presented [3,4]. The concept of integrated photovoltaics in construction was based on the direct use of PV modules in buildings as an alternative to traditional building structures such as roofing and facade systems. There are several products in the field of integrated photovoltaics. One group of materials is thin-film solar cells in the form of foil, which was described by Jelle B.P. et al. [5]. The above solution has a low deadweight, but it is not resistant to external weather conditions. This solution can be used as a facade element. Another solution is solar cells mounted on tiles or partially replacing tiles based on silicon photovoltaic cells, as described Yen-Chieh Huang et al. [6]. There are also ready-made photovoltaic modules for use on roofs, which differ from the classic use of elements that protect against weather conditions. They are most often fitted to ready-made roof solutions, as described in M. Debbarma et al. [7].

**Figure 1:**
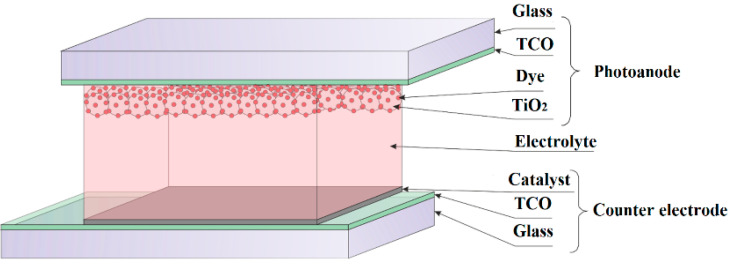
The scheme of a classical dye-sensitized solar cell.

Of the four generations of solar cells, one of the most interesting types is dye-sensitized solar cells, which use processes similar to those occurring in nature to convert solar energy into electricity (the difference is that their donor-acceptor systems transform light energy into electricity, and not into chemical energy as in photosynthesis) [8,9,10]. The working principle of such solar cells is slightly different from typical organic solar cells. A dye-sensitized solar cell consists of two glasses with transparent conductive oxide (TCO) layers as electrodes [11,12,13,14]. On the photoelectrode surface, there is an n-type semiconductor layer with a wide energy gap made of a two-component metal oxide, such as TiO_2_ and ZnO. The metal oxide is a poor photon absorber, but its association with organic dye molecules increases its conductivity. The dye is also a photosensitizer that absorbs photons of solar radiation in the range between 400–700 nm. The most commonly used dyes are organometallic compounds, including the compounds of ruthenium, osmium, and copper [13,14,15]. The electrolyte that fills the surface between the semiconductor layer and the anode is usually a solution containing the I/I^3−^ redox system. The electrolyte’s task is to transfer the electrons to the oxidized dye so that it returns to its basic state. The construction of a dye-sensitized solar cell closes the counter electrode (Figure 1). The task of the counter electrode is to collect the electrons flowing from an external current to catalyze the reduction of ions in the electrolyte. Platinum is the most commonly used material for the counter electrode [16,17,18,19,20]. The morphology of platinum and its surface roughness play a crucial role in determining the overall efficiency of dye-sensitized solar cells. Rapid progress in modern industries, including photovoltaics, depended mostly on the capabilities of the materials forming and surface engineering. The most common platinum thin films were prepared by screen printing, sputtering (PVD), or dry plasma reduction methods [21,22].

Despite the fact that the highest recorded efficiencies are noted for dye sensitized solar cells produced on a glass substrate, new substrates are sought that will allow them to be used in building integrated photovoltaics BIPV for, e.g., roof tiles or building facades, thanks to which not only windows and roofs, but also walls, will also be able to produce electricity. Replacing glass in a DSSC with other materials, such as light and flexible plastics films, metals, steel, or paper, may additionally decrease costs, because the glass substrate constitutes 15–20% of the price of a solar cell. The price of PET/ITO substrates has increased by 250% in the last 10 years, which makes it an expensive material to produce solar cells [14]. In order to replace glass with PET/ITO foil, a low-temperature heat treatment of titanium oxide was developed [23,24]. This enabled the use of a polymer film in the construction of a flexible solar cell. Stainless steel mesh was also used to obtain a flexible device. Thanks to this procedure, an efficiency of about 2.8% was obtained [25,26]. Metals foils have been increasingly used as alternative substrates for flexible DSSCs to overcome the limitations arising from the low sintering-temperature tolerance of polymer substrates [27]. However, the potential problem of metal corrosion in the electrolyte threatens to degrade the performance and long-term stability of metal-based DSSCs. For this reason it was applied nanocrystalline TiN and TiN/Ti thin film barriers on the metal substrate [28]. Dye sensitized solar cell (DSSC) on paper substrates with 1.21% efficiency was also reported [29]. Paper substrates not only offer the advantages of flexibility, portability, and lightweight, but also provide opportunities for easy implantation to textiles.

This article presents the results of research on the manufacture of innovative dye-sensitized solar cells on the surface of low-cost ceramic tiles and nickel foils. Commercial tiles are typically more porous than laboratory tiles and have a surface porosity in a size range of tens of microns. In this study, the continuous and homogeneous fluorine-doped tin oxide film was directly deposited on it and on the nickel foil. Then, nanocolloidal platinum paste was deposited as a catalytic material. The ceramic tile prepared in this way was used as the counter electrode of a dye sensitized solar cell.

## 2. Material Descriptions and Research Methodology

### 2.1. Substrates Modification

In the first stage, a layer of transparent conductive oxide in the form of fluorine-doped tin oxide (FTO) was deposited by atomization on ceramic tiles and nickel foils. All chemical reagents, FTO glass plates and nickel foil were purchased from Merck Sp. z o.o., an affiliate of Merck KGaA, Darmstadt, Germany. Ceramic roof tiles were purchased from CREATON Polska sp.z o.o. Olkusz, Poland. The thickness of the ceramic tile is 1 cm, while the nickel foil is 0.02 mm. This method is based on the deposition of a sol on the surface of a substrate using a bottle with an atomizer. The application of this method allows the temperature of the substrate to be controlled in a wider range than conventional spraying deposition methods. Moreover, deposition by atomization does not require an additional carrier gas for spraying. In the first stage, in order to produce a counter electrode, a layer of transparent conductive oxide in the form of fluorine-doped tin oxide (FTO) was deposited by an atomization method. Dibutyltin diacetate (DBTDA) was dissolved in ethanol. NH4F was dissolved in water. These two solutions were mixed and agitated ultrasonically for 30 min. This mixture was sprayed onto a heated ceramic tile and nickel foil (surface temperature = 100 °C) by the bottle with an atomizer. Then, nanocolloidal platinum paste (Sigma Aldrich) was used as a catalytic counter electrode. Platinum paste was applied to the FTO ceramic tile/nickel foil using a screen-printing method. In this article, an MS300FRO screen-printing machine was used. After printing, the wet film was dried at 125 °C for 10 min. After that, layers of platinum paste were sintered in an oven. The oven was heated from room temperature to 500 °C with a heating rate of 15 °C/min, and was maintained at 500 °C for 30 min.

### 2.2. Characterization Methods

**Figure 2:**
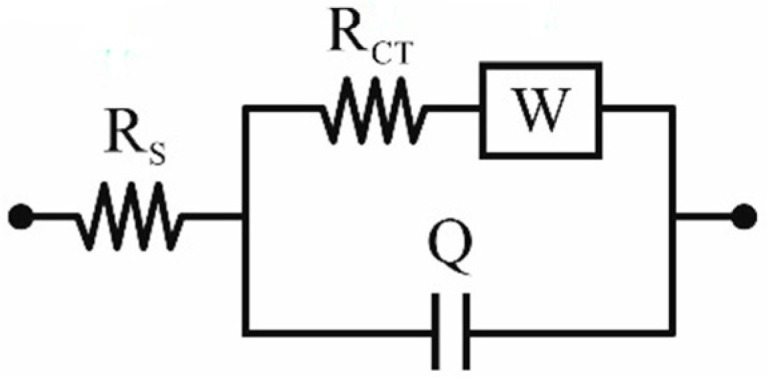
The Randles electrical equivalent system.

Scanning electron microscope (SEM) images were taken with a Supra 35 (Zeiss, Thornwood, NY, USA). The accelerating voltage was 5 kV. To obtain images of the surface topography, the secondary electron detector (by the in-lens detector) was used. Qualitative studies of the chemical composition were also performed using energy-dispersive spectrometry (EDS) (Zeiss, Thornwood, NY, USA). The thickness of the FTO films was assessed with an alpha spectroscopic ellipsometer (SE) from Woollam (Lincoln, OR, USA). The measurements were carried out at room temperature under angles of 65°, 70°, and 75°. The ψ and ∆ measurements were performed on a pure polished substrate in the first step and on a substrate with deposited thin film in the further step. The thickness value was determined with software based on the one model used. The used model included several layers (substrate/native oxide/TiO_2_/air), where the parameters of individual layers were fitted step by step (in the first step just for the substrate and in the second step for the substrate with the deposited film). The thin film of TiO_2_ was fitted with a Cauchy layer. The electrical parameters were obtained from the Hall Effect measurements. An RH2035 system operating (PhysTech GmbH, Moosburg, Germany) at a magnetic field of B = 0.426 T was used. The measurements were carried out at room temperature in the Van der Pauw mode. Further structural testing of the deposited thin films was performed using an inVia Reflex Raman spectrometer equipped with an Arion laser with a 514.5 nm length for a spectral range of 150–3200 cm^−1^ (Renishaw New Mills, UK). X-ray diffraction studies were carried out on an X’Pert Pro MPD diffractometer by PANalytical (Malvern Panalytical Ltd, Malvern, UK), using filtered (Fe filter) radiation from an X-ray tube with a cobalt anode (Co λ = 1.7909 Å), supplied with 40 kV voltage, with a filament current of 30 mA. A PIXcell 3D state detector (Malvern Panalytical Ltd., Malvern, UK) was applied on the axis of the diffracted beam. X-ray diffraction measurements were made in the Bragg-Brentano geometry in the angle range 20°–80° [2θ] with a 0.05° step and a counting time of 60 s per step. The obtained diffractograms were analyzed using X’Pert High Score Plus software v. 3.0e, together with the dedicated structural database PAN-ICSD. The results were processed by the WiRE3.1 program. The value of electric charge transfer from the counter electrode to the electrolyte was investigated using electrochemical impedance spectroscopy (EIS). The electrochemical measurements were made using a potentiostat-galvanostat coupled with an impedance meter (ATLAS 0531 EU&IA) (Atlas-Sollich, Gdańsk, Poland). The impedance spectra were recorded in the range of 0.05–105 Hz, for the alternating-current excitation amplitude *V*_ac_ of 10 mV, at an open-circuit voltage. A platinum electrode was used as the reference electrode. The EIS measurements were made in an electrolyte solution consisting of LiI (0.5 mol L^−1^), I_2_ (0.05 mol L^−1^), and 4-*tert*-butylpyridine (0.6 mol L^−1^) in acetonitrile. For the interpretation of the obtained spectra, the electric Randles equivalent system shown in Figure 2 was used, which contains the resistive element *Rs*, corresponding to the resistance of the solution (which does not depend on the catalytic layers, but on the type of electrolyte used), which is set up with two series-connected systems, namely: resistance (*R*_ct_), Warburg impedance (*W*), and a constant-phase element (*Q*). *R*_ct_ characterizes the charge transfer resistance of the counter electrode and the charge recombination resistance of the photoanode, while the Warburg impedance (*W*) relates to the ion diffusion resistance of the electrolyte. Element *Q* is the double-layer capacitance, which was used due to spatial inhomogeneities at the photoanode/electrolyte interface and surface imperfections such as the layer porosity and surface roughness.

### 2.3. Device Fabrication and Characterization

The developed solar cells consist of three elements: a photoanode, a liquid electrolyte, and a counter electrode. The counter electrode was prepared as described in the “substrates modification” section.

**Figure 3:**
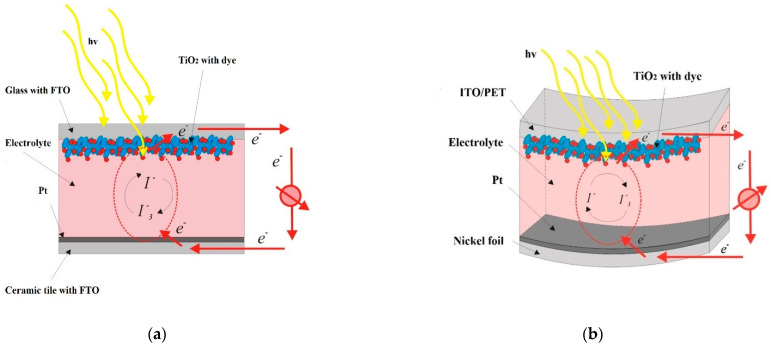
The scheme of a prepared building integrated dye sensitized solar cells with ceramic tile (**a**) and nickel foil (**b**) counter electrode.

In the next stage, in order to produce a photoanode, on the commercially-available FTO glass plate (in case of a ceramic tile) and PET/ITO (in the case of nickel foil), a titanium oxide blocking nanolayer was deposited by an atomic layer method. Then, a layer of porous nanocrystalline titanium oxide was applied by a screen printing method (18 NR-T, Greatcell Solar Materials Pty Ltd, Melbourne, Australia). The printing of TiO_2_ paste was repeated twice, obtaining a layer with a thickness of about 13 µm. Based on our experience, with this thickness of TiO_2_, we obtained the best solar cell efficiency results. The dye (N719, Merck Sp. z o.o., an affiliate of Merck KGaA, Darmstadt, Germany) was then applied by dipping the glass plates with deposited layers in an ethyl alcohol dye solution to obtain the final form of the photoanode. The active thin film was immersed in N719 dye at room temperature for 24 h. The concentration of N719 dye in EtOH was 0.25 mM. In the last stage, in order to obtain integrated dye-sensitized solar cells, the counter electrode and photoanode were joined together with the layers directed inwards, and a liquid electrolyte (EL-HSE, Merck Sp. z o.o., an affiliate of Merck KGaA, Darmstadt, Germany) containing an I^-^/I^3-^ redox couple was placed between them by spotting. The scheme of the produced DSSCs is shown in Figure 3. The dye-sensitized solar cells with an active area of 0.4 cm^2^ defined by screen mesh during screen printing method were prepared.

The electrical parameters of the manufactured DSSCs were characterized by measurements of current–voltage (I–V) characteristics using a Solar Cell I–V Tracer System (PV Test Solutions Tadeusz Zdanowicz, Wrocław, Poland) and a Keithley 2400 source meter (Tektronix, Beaverton, OR, USA) under standard AM 1.5 radiation and a light intensity of 1000 W/m^2^, according to the European standard IEC 61853-1. The intensity of incident light was calibrated by the National Renewable Energy Laboratory NREL-certified silicon reference cell equipped with KG3 filter.

## 3. Results and Discussion

**Figure 4:**
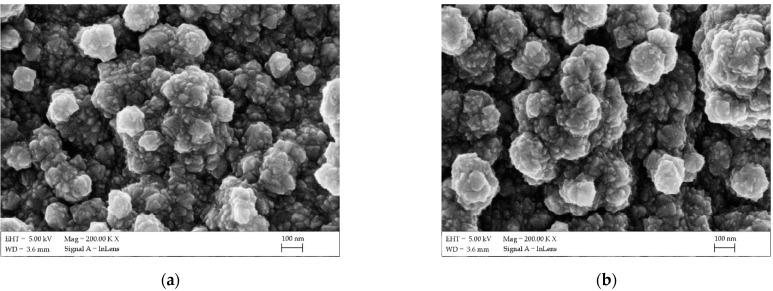
SEM surface topography images of the fluorine-doped tin oxide (FTO) sprayed thin film on the ceramic tile (**a**) and nickel foil (**b**).

SEM images were taken with a Zeiss Supra 35 using an accelerating voltage of 5 kV (Figure 4). To obtain images of the surface topography, the secondary electron (in-lens) detector was used. In both cases, an even, continuous layer with no visible defects was obtained. In turn, the application of substrate heating initiated the crystallization of the FTO layer. The grain size did not exceed 100 nm, but they combined into larger agglomerates repeated over the entire surface. The surface morphology results were similar to those available in the literature using atomized spray pyrolysis [30], but the surface roughness was much higher than that obtained by magnetron sputtering [31]. This may be due to the effect of an uneven topography of the surface of the ceramic plate. Literature data are usually based on layers deposited on glass or other polished substrates.

**Figure 5:**
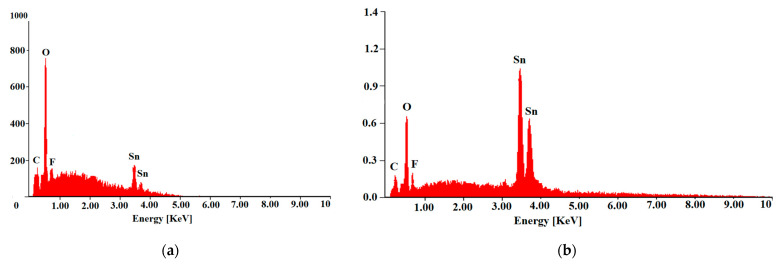
EDS spectrum of the FTO sprayed thin film on the ceramic tile (**a**) and nickel foil (**b**).

Qualitative studies of the chemical composition were also performed using energy-dispersive spectrometry (EDS), which recorded the reflections characteristic of the tin, fluorine, and oxygen from the layer (Figure 5). For tin, lines at 3.444 eV (spectrum line Lα1) and at 3.663 eV (spectrum line Lβ1) were registered. Oxygen appeared at 0.525 eV (spectrum line Kα1), and a low fluoride content was also recorded at 0.677 eV (spectrum line Kα1).

Measurement of the thickness was performed using a spectroscopic ellipsometer. The spectral range was 300–900 nm. The sampel was measured at fixed angles of incident of e of φ = 65°, 70°, and 75°. The model fit the measurement excellently. The thickness was equal to 755.43 nm.

Table 1 presents the electrical parameters of the FTO samples deposited on the ceramic tile and nickel foil. The influence of the applied substrate temperature on the electrical properties was observed. An increase in the carrier concentration and mobility was recorded. As a result, the resistivity of the deposited layers decreased. The layer deposited on the heated substrate displayed a resistivity of 5.76 × 10^−5^ Ω cm, a carrier mobility of 24.30 cm^2^/V s, and a carrier concentration of 4.54 × 10^21^ cm^−3^. The obtained resistivity was slightly lower than that reported on the layers deposited using atomized spray pyrolysis and magnetron sputtering [30,31].

**Figure 6:**
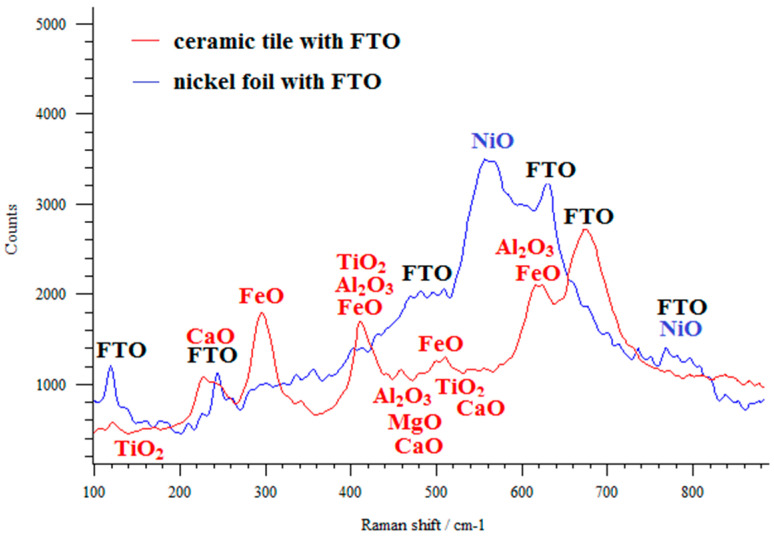
Raman spectra of the FTO sprayed thin film on the ceramic tile and nickel foil.

Raman spectrometry is a helpful tool for evaluating the quality of a prepared layer. Raman spectra make it possible to identify particles and study their structure. The Raman spectra of the FTO layer deposited on different surfaces (ceramic tile and nickel foil) were recorded (Figure 6). For the deposited FTO layer, vibration bands for SnO and SnO_2_ molecules were recorded at 115, 244, 474, 494, 561, 633, and 772 cm^−1^, which correlates with well-described results in the literature [32]. According to group theory, rutile SnO_2_ belongs to the D4h point group. The Γ point can be presented at Γ = A1g + A2g + A2u + B1g + B2g +2B1u + Eg + 3Eu. At a wavenumber of 474 cm^−1^, vibrations of oxygen were recorded (*Eg*). The bands at Raman shifts of 633 and 772 cm^−1^ correspond to the A1g and B2g vibration modes, respectively. The A1g and B2g modes are related to the vibration mode of the expansion and contraction of SnO bonds.

Products such as clay, quartz, and dyes are used to make ceramic tiles. Clay is mainly composed of silicon oxides (SiO_2_ approximately 50%); rare earth oxides (R_2_O_3_ approximately 25%); aluminum oxides (Al_2_O_3_ approximately 15%); and oxides of iron, titanium, magnesium, calcium, and potassium (approximately 10%). Therefore, ceramic tile is a product that consists of many different molecules, as shown in the Raman spectrum. The bands derived from the vibrations of SiO_2_ were recorded at 321, 335, 354, 393, and 454 cm^−1^ wavelengths. Rare earth molecules, depending on their composition, produce bands in the range of 95–660 cm^−1^. The vibrations of aluminum oxide molecules produce bands at 360, 400, 460, 610, and 620 cm^−1^. Bands originating from the vibrations of iron oxide molecules were recorded at 220, 300, 410, 500, and 620 cm^−1^. At 150, 400, 520, and 645 cm^−1^, bands from the vibrations of titanium dioxide molecules were also recorded. The vibrations of the MgO molecule produced bands at 420, 476, and 594 cm^−1^. Small bands from the vibrations of the CaO molecule at 464, 522, and 553 cm^−1^ were also recorded. The typical signals for the NiO_x_ layer on the Ni metal surface were recorded—bands due to the stretching mode of the NiO molecule were recorded at 540 and 800 cm^−1^.

**Figure 7:**
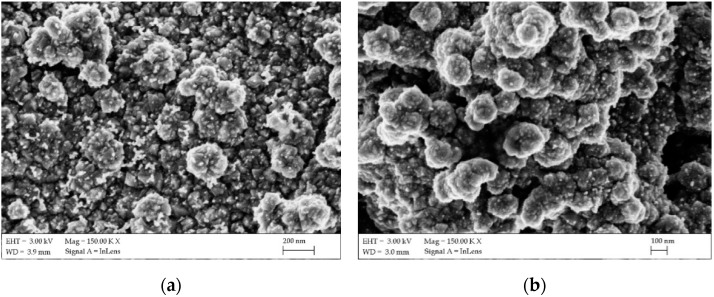
SEM surface topography images of the platinum on the FTO/ceramic tile (**a**) and FTO/nickel foil (**b**).

**Figure 8:**
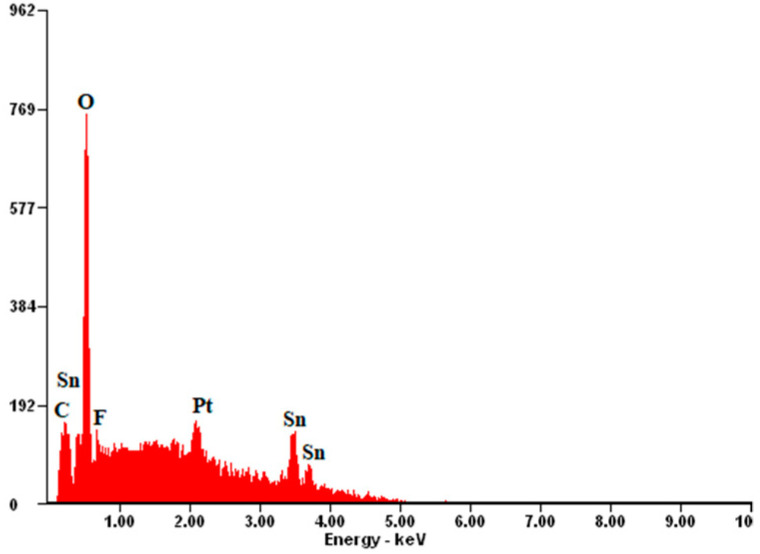
EDS spectrum of platinum on the FTO/ceramic tile substrate.

Platinum paste was applied on an FTO ceramic tile using a screen-printing method. The layer thickness was about 0.5 µm. In this article, a screen-printing machine (MS300FRO) was used. After printing, the wet film was dried at 125 °C for 10 min, and two more layers were applied. After that, three layers of platinum paste were sintered in an oven. The oven was heated from room temperature to 500 °C with a heating rate of 15 °C/min, and was maintained at 500 °C for 30 min. Platinum tends to increase the so-called “islands” (Figure 7). The figures document the structure of the FTO with deposited platinum islands, which correlates with the results known from the literature [33]. A greater packing density was recorded on the FTO deposited on the ceramic tile substrate. To confirm that the observed thin films are platinum, the qualitative chemical composition was studied using EDS spectra, and the reflections typical for platinum at 2.127 keV (spectrum line Mβ1) were registered (Figure 8).

**Figure 9:**
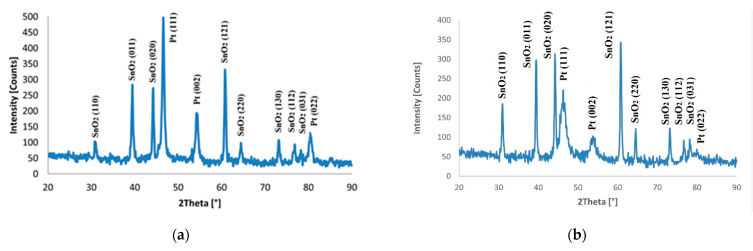
The diffraction pattern of screen-printed platinum thin film deposited on the FTO/ceramic tile (**a**) and FTO/nickel foil (**b**).

To analyze the diffractogram, JCPDS files were used, according to which the appropriate Miller indices were assigned. Platinum thin films were characterized by a constant angle of incidence identifying the characteristic peaks of platinum and tin oxide. The crystal structure for platinum was identified as face-centered cubic based on card no. 98-018-3075 (Figure 9).

**Figure 10:**
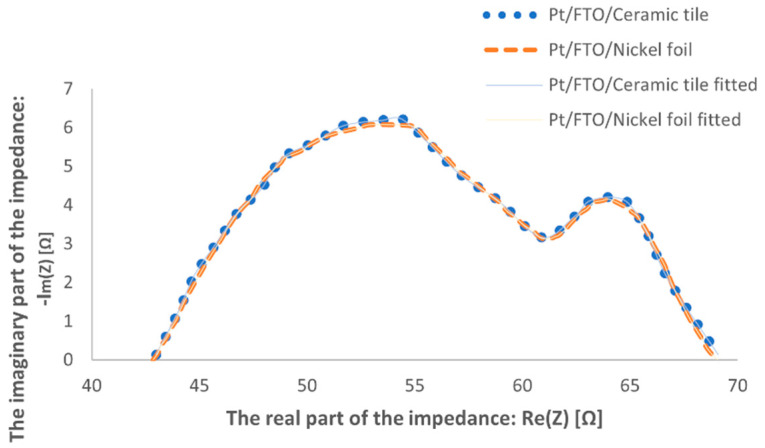
The electrochemical impedance spectroscopy (EIS) spectra of screen-printed platinum thin films deposited on the FTO/ceramic tile and FTO/nickel foil.

In the Nyquist diagram of the layers used as the counter electrodes of dye-sensitized solar cells, two semicircles overlapped each other in time becaue of their similar time constants (Figure 10). The platinum layers deposited on the FTO/ceramic tile and FTO/nickel foil had similar charge transfer resistance values of 27.3 and 26.5 Ω, respectively (Table 2).

**Figure 11:**
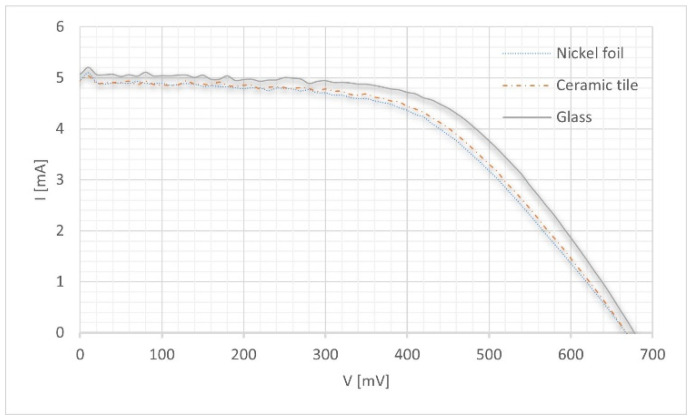
Current−voltage characteristics of experimental dye sensitized solar cell (DSSC).

The electrical parameters of the manufactured DSSCs were characterized by measuring the current−voltage (I−V) characteristics using a PV Test Solutions Tadeusz Zdanowicz Solar Cell I−V Tracer System and a Keithley 2400 source meter under standard AM 1.5 radiation. It can be seen that the shape of the current−voltage characteristic of the solar cell with a ceramic tile counter electrode was less rectangular than the solar cell with a glass counter electrode. It can be seen (Figure 11) that the nickel foil and tile substrates had a slight influence on the short-circuit current and open-circuit voltage, which consequently affected the efficiency of the dye-sensitized solar cells. Under the same illumination conditions, the Voc values of the DSCs with glass electrode were higher than that of the DSC with ceramic tile and nickel foil electrodes. Additionally, the values of charge transfer resistance Rct were greater than the values described in the literature [34,35], and can be directly related to a lower Voc. These electrodes showed a low charge-transfer resistance because of their large surface area.

The basic electrical parameters of the measured solar cells are presented in Table 3. An analysis of the measured electrical parameters showed that the cells with a ceramic tile had a higher efficiency than the cells with nickel foil from among the produced integrated solar cells. The rough surface of the ceramic tile and the bent nickel foil may result in a slightly lower efficiency (than in the case of cells with a glass counter electrode). This could affect, in particular, the contact between the probe and the electrode.

## 4. Conclusions

Building-integrated dye-sensitized solar cells were manufactured based on ceramic tile and nickel foil substrates. The advantage of this solution is a high level of integration with construction, while requiring the same time to generate electricity. To produce a counter electrode, a layer of transparent conductive oxide in the form of fluorine-doped tin oxide (FTO) was deposited using an atomization method. The crystalline structure of the atomized FTO was recorded. Then, a platinum paste was applied on the FTO tile and foil using a screen-printing method to construct a counter electrode. The SEM studies confirmed that a layer of FTO was deposited without major contamination or damage, and the grain size did not exceed 100 nm. The electrical properties of the dye-sensitized solar cells with a ceramic tile and foil as the counter electrode were the highest, which was close to the efficiency of a solar cell using conventional glass as the counter electrode. Such electrodes demonstrate the possibility of fabricating stable, low-cost, and effective building-integrated dye-sensitized solar cells.

## Figures and Tables

**Table 1:** Electrical parameters of the investigated FTO layers: resistivity (*ρ*), carrier concentration (*n*), and carrier mobility (*µ*).

FTO Samples	*ρ* [Ω·cm]	*n* [cm^−3^]	*µ* [cm^2^/V·s]
Nickel foil (non heated)	1.23 × 10^−4^	2.68 × 10^21^	24.20
Nickel foil (Heated = 100 °C)	5.69 × 10^−5^	4.52 × 10^21^	24.30
Ceramic tile (non heated)	1.26 × 10^−4^	2.71 × 10^21^	24.10
Ceramic tile (Heated = 100 °C)	5.76 × 10^−5^	4.54 × 10^21^	24.30

**Table 2:** Values of charge transfer resistance *R*_ct_ and solution resistance *R*_s_ from the EIS spectra of the produced counter electrodes.

Pt Samples	*R_ct_* [Ω]	*R_s_* [Ω]
Nickel foil	26.5	42.5
Ceramic tile	27.3	41.8

**Table 3:** Electrical parameters of the experimental DSSCs.

DSSC Sample	Active Area [cm^2^]	*J_sc_* [mA/cm^2^]	*I_sc_* [mA]	*V_oc_* [mV]	*I_max_* [mA]	*V_max_* [mV]	*P_max_* [mW]	*FF*	*E_ff_* [%]
Nickel foil	0.4	12.40	4.959	667.453	4.157	425.694	1.769	0.53	4.34
Ceramic tile	0.4	12.36	4.944	668.392	4.215	431.203	1.818	0.55	4.48
Glass	0.4	12.68	5.072	677.769	4.395	450.470	1.980	0.58	4.83

## Data Availability

Not applicable.
